# Beyond Benign: A Case of Subependymal Giant Cell Astrocytomas Provoking Hydrocephalus in Tuberous Sclerosis Complex

**DOI:** 10.15388/Amed.2024.31.1.9

**Published:** 2024-02-27

**Authors:** Antonio Navarro-Ballester, Rosa Álvaro-Ballester, Miguel Ángel Lara-Martínez

**Affiliations:** Radiology Department, Hospital General Universitari de Castelló, Castellón de la Plana (Castellón), Spain

**Keywords:** Glioma, Tuberous sclerosis, Hydrocephalus, glioma, tuberozinė sklerozė, hidrocefalija

## Abstract

22-year-old male diagnosed with Tuberous Sclerosis Complex (TSC), a genetic disorder characterized by benign tumors in various organs, with a focus on neurological implications. Central to the study is the development of Subependymal Giant Cell Astrocytomas (SEGAs), leading to hydrocephalus in the patient. The diagnosis of TSC was made in the patient’s childhood, and he was monitored regularly. The study highlights a significant growth in a subependymal nodule, leading to monoventricular hydrocephalus. MRI scans played a crucial role in identifying the progression of SEGAs and the subsequent hydrocephalus. The treatment approach involved endoscopic surgical removal of the SEGA, with histopathology confirming the diagnosis. Post-surgical outcomes over an eight-year follow-up period showed a normalization in ventricular size and the stability of other subependymal nodules, without any complications. This case underscores the importance of regular monitoring for TSC patients, early intervention for complications like hydrocephalus, and the need for a multidisciplinary treatment approach. The case study provides valuable insights into the management of neurodevelopmental disorders and the complexities surrounding TSC and SEGAs.

## Abstract (it)

22 metų vyrui diagnozuotas tuberozinės sklerozės kompleksas (TSC) – genetinis sutrikimas, kuriam būdingi nepatogeniniai įvairių organų navikai, daugiausia dėmesio skiriant neurologinėms pasekmėms. Pagrindinė tyrimo tema - subependiminių gigantinių ląstelių astrocitoma (SEGA), dėl kurios pacientui išsivystė hidrocefalija. TSC diagnozė buvo nustatyta pacientui vaikystėje, jis buvo reguliariai stebimas. Tyrime išryškėja žymus subependiminio mazgelio augimas, sukėlęs monoventrikulinę hidrocefaliją. MRT tyrimas atliko lemiamą vaidmenį nustatant SEGA progresavimą ir vėliau atsiradusią hidrocefaliją. Gydymo metodas apėmė endoskopinį chirurginį SEGOS pašalinimą, o diagnozę patvirtino histopatologinis tyrimas. Pooperaciniai rezultatai per aštuonerių metų stebėjimo laikotarpį parodė, kad skilvelio dydis normalizavosi, o kiti subependiminiai mazgai išliko stabilūs ir nekilo jokių komplikacijų. Šis atvejis pabrėžia, kaip svarbu reguliariai stebėti TSC sergančius pacientus, anksti įsikišti dėl tokių komplikacijų, kaip hidrocefalija, ir taikyti daugiadisciplininį gydymo metodą. Atvejo tyrimas suteikia vertingų įžvalgų apie neurologinių raidos sutrikimų gydymą ir su TSC ir SEGA susijusius sudėtingus klausimus.

## Background

Subependymal giant cell astrocytomas (SEGAs) are rare brain tumors associated with tuberous sclerosis complex (TSC), an autosomal dominant genetic disorder characterized by the development of benign tumors in multiple organs, including the brain, skin, kidneys, heart, eyes, and lungs. The neurological manifestations of TSC are particularly significant and include the development of cortical tubers, subependymal nodules, and SEGAs. SEGAs are found in approximately 5–15% of individuals with TSC [1,2] and are typically located near the foramen of Monro, a key area for cerebrospinal fluid (CSF) flow in the brain.

SEGAs are thought to arise from subependymal nodules, which are typically asymptomatic. The transformation into SEGAs is not fully understood but is believed to be associated with mutations in either the TSC1 or TSC2 genes, which are responsible for the regulation of the mTOR (mammalian target of rapamycin) pathway [3]. Dysregulation of this pathway leads to uncontrolled cell growth and proliferation, contributing to tumor development.

SEGAs, although benign, can cause significant morbidity due to their location and potential to grow. They are often slow-growing but can become symptomatic as they enlarge, especially when they obstruct the flow of CSF, leading to obstructive hydrocephalus. Hydrocephalus in the context of TSC and SEGA can be a medical emergency, requiring prompt diagnosis and intervention [4].

The diagnosis of SEGAs typically involves a combination of clinical evaluation and imaging techniques. For patients with TSC, a high index of suspicion for SEGAs is necessary, especially when new neurological symptoms appear. Magnetic Resonance Imaging (MRI) is the gold standard for diagnosing SEGAs. These tumors appear as contrast-enhancing lesions near the foramen of Monro on MRI scans. MRI not only helps in identifying the presence of SEGAs but also in assessing their size, growth rate, and potential for causing hydrocephalus. Regular neurological assessments are crucial for TSC patients. Any new onset of symptoms such as headaches, nausea, visual disturbances, or changes in mental status might indicate tumor growth or hydrocephalus. Although not used for diagnosing SEGAs directly, genetic testing for mutations in TSC1 and TSC2 genes can confirm the diagnosis of TSC and provide valuable information for family counseling.

The rationale for publishing this case report is multifold. First, it provides insight into the rare occurrence of a SEGA causing hydrocephalus in a patient with TSC, contributing to the understanding of this complex condition. Second, it highlights the importance of regular monitoring and early intervention in patients with TSC to prevent complications such as hydrocephalus. Third, this case illustrates the challenges in managing these patients, emphasizing the need for a multidisciplinary approach.

## Case report

**Figure 1:**
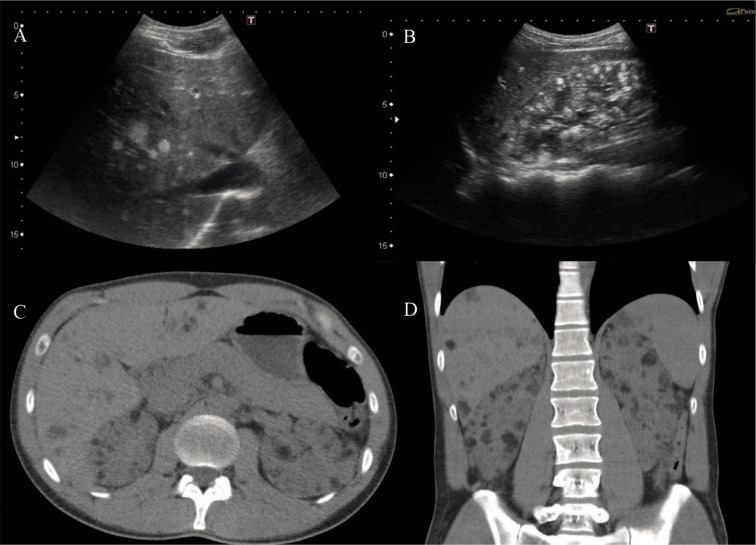
Images A and B: Ultrasonographic study showing multiple well-defined hyperechoic focal lesions diffusely distributed throughout the hepatic (A) and renal (B) parenchyma. Images C and D: Abdominopelvic CT without contrast, axial (C) and coronal (D) cuts, where multiple hypodense focal lesions with fat attenuation (< -20 UH) are visualized, diffusely distributed throughout the hepatic and renal parenchyma. Lesions are consistent with angiomyolipomas.

A 22-year-old male patient, diagnosed with TSC in childhood, visited our hospital for a biannual check-up at the Neurosurgery unit. His medical history includes hypothyroidism and obesity. He was on oral medication for occasional epileptic seizures: sodium valproate 500 mg (2 tablets every 12 hours), perampanel 8 mg (1 tablet daily), and oxcarbazepine 600 mg (3.5 tablets daily). Abdominal radiological studies showed hepatic and renal lesions with macroscopic fat, consistent with angiomyolipomas (Figure 1).

**Figure 2:**
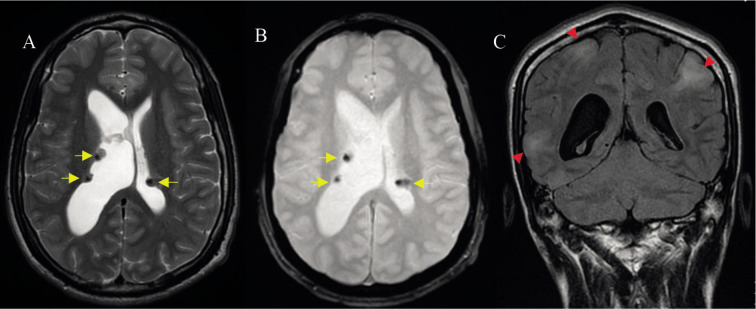
Brain MRI study. Image A and B: Axial T2 cut (A) and gradient (B) where the subependymal nodules (yellow arrows) in both lateral ventricles are identified with a drop in signal intensity in the gradient sequence due to calcification. Image C: Coronal T2 FLAIR cut, where several hyperintense cortical-subcortical lesions compatible with cortical tubers are identified (red head arrows).

**Figure 3:**
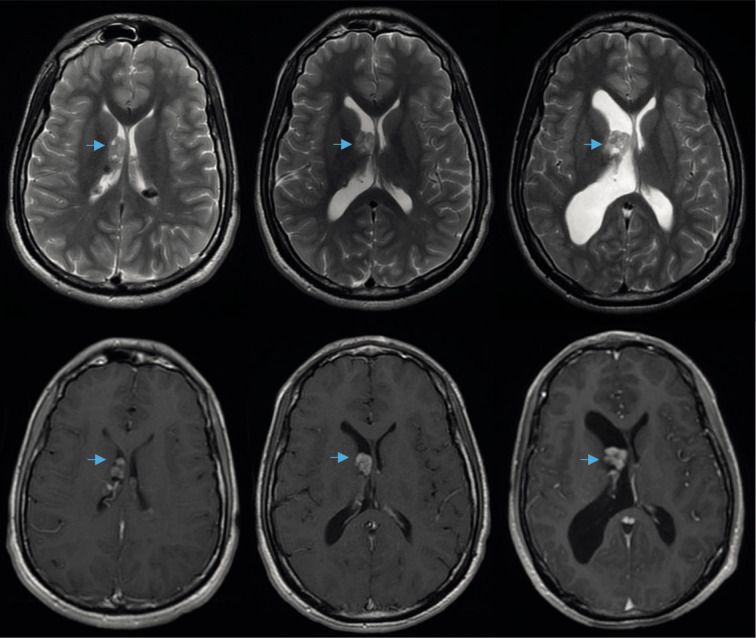
Brain MRI study from successive biannual controls. The upper images are axial T2 cuts, and the lower corresponding images are in T1 sequence with gadolinium. There is an identified enhancement with progressive increase in the size of the subependymal nodule (blue arrows) and the progressive enlargement of the right lateral ventricle.

In the brain MRI, multiple cortical-subcortical hyperintense lesions in T2 FLAIR (Fluid attenuated inversion recovery) were identified, compatible with cortical tubers and multiple subependymal nodules, some calcified, in both lateral ventricles (Figure 2). Comparing with previous studies, an increase in size of a subependymal nodule at the anterior aspect of the right lateral ventricle adjacent to Monro’s foramen was observed, associated with an increase in the size of the right lateral ventricle causing monoventricular hydrocephalus (Figure 3).

**Figure 4:**
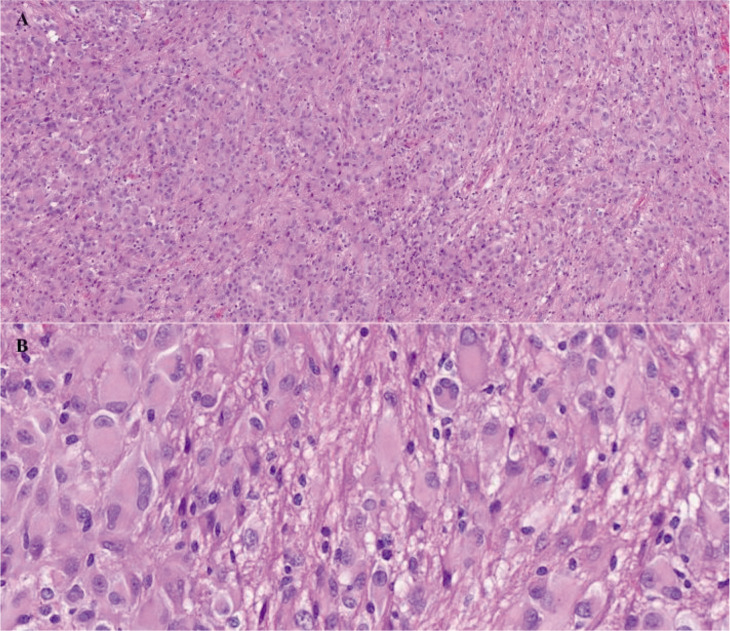
Hematoxylin-eosin stained histological section of the subependymal giant cell astrocytoma (A: image at 10x magnification and B: image at 40x magnification). There is a prominent cellular proliferation with multinucleated giant cells, interposed fibrillary septa, and a stroma rich in vessels. The cells exhibit large and pleomorphic nuclei with eosinophilic cytoplasm.

Finally, due to the growth of the subependymal nodule and the onset of right monoventricular hydrocephalus, a surgery for open endoscopic excision of the lesion was performed. The histopathological examination of the surgical specimen confirmed the clinical suspicion of SEGA (Figure 4).

**Figure 5:**
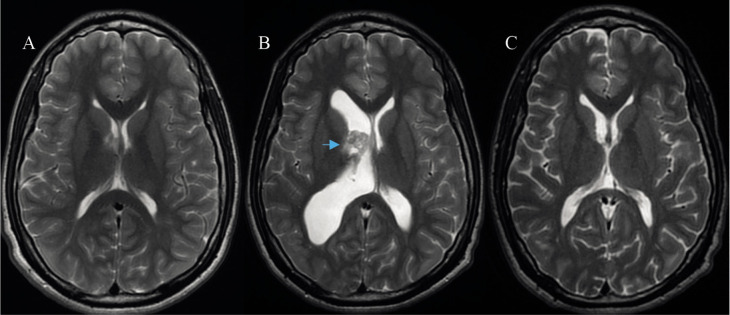
Brain MRI study, axial T2 cuts. Image A: Initial study showing normal ventricular size. Image B: Four years later compared to Image A, showing the presence of right monoventricular hydrocephalus and SEGA (blue arrow). Image C: Postoperative study displaying complete resection of the lesion and normalization of ventricular size.

Subsequent radiological controls after total resection of the lesion showed normalization of the size of the right lateral ventricle and stability of the remaining subependymal nodules (Figure 5). The patient had no post-surgical complications and remained asymptomatic in follow-ups over the next 8 years.

## Discussion

TSC is a genetic disorder that significantly impacts both morbidity and mortality. Characterized by the growth of benign tumors in various organs, TSC’s severity varies widely among individuals. Major morbidity factors include neurological complications such as epilepsy, which is common and can be difficult to control, and intellectual disability. Renal complications, like angiomyolipomas, can also lead to significant health issues. Mortality in TSC is often related to these neurological and renal complications, as well as potential cardiac issues due to cardiac rhabdomyomas [5].

Similar to our patient, 83.6% of TSC patients experience epilepsy, typically with onset in early childhood. There is a diverse range of seizure types associated to this disease. Additionally, in TSC, the presence of various seizure types presents significant management challenges, since there is a notable correlation between the control of these seizures and the degree of intellectual disability in TSC patients [6].

At The 2012 International Tuberous Sclerosis Complex Consensus, experts redefined the diagnostic criteria for TSC-related brain abnormalities as follows [7]:

1. The identification of tubers, other cortical dysplasias (like cortical migration lines), SENs or SEGAs are each classified as major criteria. The presence of any two of these criteria confirms a TSC diagnosis, consistent with the 1998 guidelines.
2. For diagnostic purposes, SEGAs are defined as lesions at the caudothalamic groove larger than 1 cm in any dimension, or any subependymal lesion exhibiting growth over time in serial imaging, regardless of its size. While most SEGAs demonstrate significant contrast enhancement, a growing subependymal lesion should be considered a SEGA even without enhancement.

SEGAs, benign brain tumors associated with TSC, have a significant link with the development of hydrocephalus. This relationship stems from the location and growth patterns of SEGAs. Typically, these tumors arise near the ventricles of the brain, which are critical pathways for the circulation of CSF. As SEGAs grow, they can obstruct these pathways, leading to an accumulation of CSF and increased intracranial pressure, a condition known as hydrocephalus.

The treatment of SEGAs, especially when complicated by hydrocephalus, requires a multidisciplinary approach, involving neurologists, neurosurgeons, and other specialists. Surgical removal of the tumor is often the primary treatment for SEGAs. The surgery aims to completely resect the tumor, alleviate symptoms, and restore normal CSF flow. In cases where complete resection is not possible, debulking of the tumor might be performed to relieve hydrocephalus. If hydrocephalus is present, it may require additional surgical intervention that includes Endoscopic Third Ventriculostomy (ETV) (This procedure creates a new pathway for CSF flow, bypassing the obstruction caused by the tumor) and a ventriculoperitoneal shunt. (In some cases, especially if ETV is not feasible, a shunt may be placed to divert CSF from the brain to another part of the body, usually the abdomen, where it can be absorbed.) Recent advances have introduced mTOR inhibitors (e.g., Everolimus) as a treatment option for SEGAs (7, 8). These drugs can reduce the size of SEGAs and are particularly useful in cases where surgery is not feasible or as an adjunct to surgery. They work by targeting the mTOR pathway, which is often dysregulated in TSC.

Post-treatment: Patients require regular neuroimaging to monitor for tumor recurrence or regrowth and to assess the effectiveness of the treatment. Supportive care: Management of TSC and SEGAs also involves supportive care, addressing other symptoms and complications of TSC, and ensuring a good quality of life for the patient.

This case exemplifies the intricate challenges in managing TSC and SEGAs, underscoring the critical role of timely diagnosis, the necessity for a multidisciplinary treatment approach, and the importance of balancing surgical and medical management strategies. It highlights the nuanced decision-making process in treating complex neurodevelopmental disorders, demonstrating how individual patient needs dictate tailored treatment plans, and emphasizes the significant role of neuroimaging in ongoing post-treatment evaluation and monitoring.
